# Radical curative efficacy of tafenoquine combination regimens in *Plasmodium cynomolgi*-infected Rhesus monkeys (*Macaca mulatta*)

**DOI:** 10.1186/1475-2875-10-212

**Published:** 2011-07-29

**Authors:** Geoffrey S Dow, Montip Gettayacamin, Pranee Hansukjariya, Rawiwan Imerbsin, Srawuth Komcharoen, Jetsumon Sattabongkot, Dennis Kyle, Wilbur Milhous, Simon Cozens, David Kenworthy, Anne Miller, Jim Veazey, Colin Ohrt

**Affiliations:** 1 Division of Experimental Therapeutics, Walter Reed Army Institute of Research, 503 Robert Grant Ave, Silver Spring, MD 20910, USA; 2 Department of Veterinary Medicine, United States Army Medical Component, Armed Forces Research Institute of Medical Sciences, 315/6, Rajthevi, Bangkok, 10400, Thailand; 3 Department of Entomology, United States Army Medical Component, Armed Forces Research Institute of Medical Sciences, 315/6, Rajthevi, Bangkok, 10400, Thailand; 4 College of Public Health, University of South Florida, 3720 Spectrum Blvd, FL 33612, USA; 5 GlaxoSmithKline R&D, Drug Metabolism and Pharmacokinetics, Park Road, Ware, Hertfordshire, SG12 0DP, UK; 6 CPMS, QSi, GlaxoSmithKline, Mail Cose UW2431, 709 Swedeland Road, King of Prussia, PA, 19406, USA; 7 US Army Medical Material Development Activity MCMR-UMP 1430 Veterans Drive Fort Detrick, MD 21702-9232, USA

## Abstract

**Background:**

Tafenoquine is an 8-aminoquinoline being developed for radical cure (blood and liver stage elimination) of *Plasmodium vivax*. During monotherapy treatment, the compound exhibits slow parasite and fever clearance times, and toxicity in glucose-6-phosphate dehydrogenase (G6PD) deficiency is a concern. Combination with other antimalarials may mitigate these concerns.

**Methods:**

In 2005, the radical curative efficacy of tafenoquine combinations was investigated in *Plasmodium cynomolgi*-infected naïve Indian-origin Rhesus monkeys. In the first cohort, groups of two monkeys were treated with a three-day regimen of tafenoquine at different doses alone and in combination with a three-day chloroquine regimen to determine the minimum curative dose (MCD). In the second cohort, the radical curative efficacy of a single-day regimen of tafenoquine-mefloquine was compared to that of two three-day regimens comprising tafenoquine at its MCD with chloroquine or artemether-lumefantrine in groups of six monkeys. In a final cohort, the efficacy of the MCD of tafenoquine against hypnozoites alone and in combination with chloroquine was investigated in groups of six monkeys after quinine pre-treatment to eliminate asexual parasites. Plasma tafenoquine, chloroquine and desethylchloroquine concentrations were determined by LC-MS in order to compare doses of the drugs to those used clinically in humans.

**Results:**

The total MCD of tafenoquine required in combination regimens for radical cure was ten-fold lower (1.8 mg/kg *versus *18 mg/kg) than for monotherapy. This regimen (1.8 mg/kg) was equally efficacious as monotherapy or in combination with chloroquine after quinine pre-treatment to eliminate asexual stages. The same dose of (1.8 mg/kg) was radically curative in combination with artemether-lumefantrine. Tafenoquine was also radically curative when combined with mefloquine. The MCD of tafenoquine monotherapy for radical cure (18 mg/kg) appears to be biologically equivalent to a 600-1200 mg dose in humans. At its MCD in combination with blood schizonticidal drugs (1.8 mg/kg), the maximum observed plasma concentrations were substantially lower than (20-84 *versus *550-1,100 ng/ml) after administration of 1, 200 mg in clinical studies.

**Conclusions:**

Ten-fold lower clinical doses of tafenoquine than used in prior studies may be effective against *P. vivax *hypnozoites if the drug is deployed in combination with effective blood-schizonticidal drugs.

## Background

Tafenoquine has potential to be a very important tool in malaria elimination efforts. The 8-aminoquinoline class is the only class that kills key survival stages of the parasite - *Plasmodium vivax *hypnozoites and stage 5 *Plasmodium falciparum *gametocytes. It is currently in Phase II/III clinical trials for the radical cure of *P. vivax *malaria, in a one-day regimen as a consequence of its long half-life [1]. In two recent case reports, tafenoquine was administered alone to two patients with acute *P. vivax *malaria instead of the normal sequential combination of chloroquine (1500 mg over three days) and primaquine (420 mg over 14 days, [2]). No relapses or recrudescences were observed in these two cases. However, parasite clearance times (approximately 84 h) were longer than those observed for typical blood schizonticidal drugs. Tafenoquine, like other 8-aminoquinolines such as primaquine, induced haemolytic anaemia in individuals with glucose-6-phosphate dehydrogenase (G6PD) deficiency [3,4]. Human studies to assess this risk are on-going.

Appropriate combination of tafenoquine with a more potent and fast acting blood schizonticidal drug (effective against blood-stage malarial parasites) has the potential to yield a more rapidly acting regimen. Also, if such combination allowed the dose of tafenoquine required for radical cure to be reduced, its haemolytic toxicity at therapeutic doses could be mitigated. Chloroquine, mefloquine, artemether-lumefantrine and other forms of artemisinin-based combination therapy (ACT) would be logical blood schizonticidal partner drugs. Chloroquine is already the standard cure for blood-stage treatment of symptomatic *P. vivax *malaria in many parts of the world. The combination of tafenoquine and chloroquine is synergistic against chloroquine-resistant *P. vivax *in *Aotus *monkeys [5]). Mefloquine and piperaquine would be good pharmacokinetic matches for tafenoquine based on their long half-lives [6]. ACT offers an advantage over mefloquine and chloroquine due to its activity against drug-resistant *P. falciparum *[7].

There is not currently an animal model of *P. vivax *available to evaluate the effects of drugs on the blood and liver stages of *P. vivax*. Traditionally, the *Plasmodium cynomolgi*-Rhesus monkey relapsing malaria model [8,9] has been used as a surrogate and has been predictive of effects for the human *P. vivax *treatment. The USAMC-AFRIMS first used the Indian-origin Rhesus monkey/*P. cynomolgi *malaria model for anti-malarial drug testing in 1975 [10]. The infection is detected readily by visualizing the parasites on blood smears. Rhesus monkeys develop parasitaemia to a greater magnitude and uniformity than cynomolgus monkeys which demonstrated self cure [11]. Rhesus monkeys with an intact spleen usually tolerate the infection very well and show no signs of clinical illness. This model is an important tool in the development of drugs to treat *P. vivax *malaria.

In this study, this model was utilized to determine whether the monotherapy dose of tafenoquine required for radical cure could be reduced by combination with chloroquine. The utility of mefloquine, atovaquone-proguanil and artemether-lumefantrine as partner drugs for tafenoquine was also assessed relative to chloroquine.

## Methods

The United States Army Medical Component, Armed Forces Research Institute of Medical Sciences (USAMC-AFRIMS) Institutional Animal care and Use Committee (IACUC) and the Animal Use Review Division, U.S. Army Medical Research and Materiel Command reviewed and approved these studies. Animals were maintained in accordance with established principles under the *Guide for the Care and Use of Laboratory Animals *[12]. The USAMC-AFRIMS animal care and use programme has been considered an exemplary accredited programme by Association for Assessment and Accreditation for Laboratory Animal Care (AAALAC) International.

### General methods

The radical curative effects of the drugs were evaluated in three cohorts 20, 20 and 14 *P. cynomolgi-bastianelli*-infected *Macaca mulatta *(captive bred, Indian origin, male or female, 3-6 kg, 2-4 years old, specific pathogen free, Rhesus monkeys) using methods described previously by Schmidt *et al *[8] and others [9,13-15], with the following minor adjustments from Corcoran [14] in place since 2005: (i) the use of monkey plasma to dilute the sporozoite suspension prior to iv inoculation was discontinued (ii) phosphate-buffered saline (PBS) was used to dilute the final sporozoites preparation and this was later supplemented with 5% bovine serum albumin, (iii) phase-contrast microscopy was used to quantify active sporozoites from freshly harvested mosquito salivary gland suspension rather than lengthy staining methods, and (iv) the inoculum was standardized as a single intravenous injection of one million sporozoites per monkey. The sporozoites were obtained from salivary gland dissection of 100-300 *Anopheles dirus *mosquitoes that had been fed 14-16 days earlier on a splenectomized donor monkey bearing the malarial gametocytes in its peripheral blood.

Once the experimental monkeys were inoculated, Giemsa-stained blood smears were prepared from peripheral blood samples, and parasitaemia results were determined daily from days 6 to 21 post-inoculation. Monkeys were administered study drugs once patency was confirmed, and parasitaemia reached 5,000/μl for 1-3 days per os (PO) as outlined in Table 1 and Additional File 1 and the remainder of this section. After study day 21, if blood smears remained negative, they were monitored three days per week for four weeks and then twice weekly until 100 days post treatment, after which the animals were considered to be radically cured. In this model if treatment failure occurs, the animals relapse multiple times but in the interests of practicality only the 1^st ^and 2^nd ^relapses are treated with new anti-malarial compounds. Blood smears were monitored daily for 21 days, then three times for four weeks, and twice weekly until 100 days post treatment. At the first and second relapses, monkeys were treated with different combinations of study drug orally as appropriate. They were administered a curative dose of standard (expected to be 100% radically curative based on legacy data) radical curative drug regimen, chloroquine/primaquine (10/1.78 mg/kg/day PO for seven days) as indicated below at the third relapse or earlier if additional study drugs were not given. Based on institutional legacy data, this dose is known to be associated with 100% radical cure.

**Table 1:** Outcome of Chloroquine Treatments in Various Cohorts

Cohort#	Chloroquine Regimen	Relapse #	Number of Cleared or Cured Infections	Average Time To Relapse From Termination of Treatment in Days (+/- Standard Deviation)
2&3	24 mg/kg/day for three days	Primary Treatment	4 of 4	9.8 +/- 1.7
1-3	24 mg/kg/day for three days	Second Relapse	6 of 6	12.7 +/- 0.8
1-3	24 mg/kg/day for three days + PQ	Second or Third Relapse	9 of 9 radically cured	No relapses

Tafenoquine was obtained from GlaxoSmithKline (Research Triangle Park, North Carolina). Primaquine were obtained from the Walter Reed Army Institute of Research (WRAIR) Chemical Inventory System. Both compounds were administered as a suspension in 0.5% hydroxyethylcellulose (HEC) vehicle. Chloroquine diphosphate was obtained from Sigma and administered in HEC. Pre-formulated quinine dichloride was obtained from ANB Laboratories, Thailand, and was administered intramuscularly. Artemether-lumefantrine (Novartis) tablets were obtained commercially and homogenized in an appropriate volume of HEC for oral administration. All oral drugs were dispensed by disposable paediatric intra-gastric feeding tubes. During the period of drug administration and follow-up the animals were housed in individual cages and regular enrichment items were offered. They were fed a fixed ration of biscuits supplemented with enrichment items including fresh fruits and seasonable vegetables and routinely monitored up to three times daily for any changes in activity, food consumption or other clinical signs. The animals were weighed regularly during the study period. Relevant clinical laboratory data were generated prior to, daily through the initial treatment period and weekly thereafter. Except where indicated, all changes in body weight, general health or clinical laboratory values were consistent with onset and recovery from malaria infection, and are only reported here where they appeared to be related to an effect of study drugs.

### Radical curative effects of tafenoquine at various doses alone and in combination with chloroquine (Cohort 1)

This cohort objective was to identify the minimum dose of tafenoquine required, alone, or in combination with chloroquine, to radically cure *P. cynomolgi *malaria in Rhesus monkeys. In this context, radical cure is defined as the elimination of blood and liver stage parasites. Groups of two monkeys received tafenoquine at doses of 0.2, 0.6, 2 and 6 mg/kg/day × 3 days (monotherapy) or tafenoquine at 0.06, 0.2, 0.6 and 2 mg/kg/day × 3 days combined with chloroquine at 16 mg/kg/day × 3 days, or tafenoquine at 12 mg/kg/day (single dose, monotherapy), and one chloroquine control group at 16 mg/kg/day × 3 days.

The tafenoquine doses were chosen to evenly bracket what were thought to be the ED50s of tafenoquine alone and in combination with chloroquine based on prior unpublished studies (these unpublished pilot relapse studies were conducted with either 7-day chloroquine regimens, or regimens of tafenoquine administered for one or seven days). The rationale for the three-day regimen was as follows: The standard human treatment dose for chloroquine is either 1500 mg over three days or 25 mg/kg over three days [2,16]. The conversion factor between humans and Rhesus monkeys for chloroquine on a mg/kg basis based on similar efficacy outcomes is approximately two [9]. The Rhesus monkey chloroquine dose equivalent of a human treatment dose is therefore approximately 50 mg/kg over three days. Therefore a dose of 48 mg/kg in evenly divided doses over three days was selected.

The minimum curative dose was defined as the lowest dose of tafenoquine required to cure two of two monkeys. Treatment outcome of the monotherapy regimen was defined as an early treatment failure (for blood schizonticidal activity) if the parasitaemia did not decline within the first 48 hours. In these instances, chloroquine was subsequently administered at a dose of 16 mg/kg/day for three days. In the tafenoquine alone groups, relapses were treated with the next highest dose of tafenoquine. In the combination groups, relapses were treated with the same dose of tafenoquine and an adjusted higher dose of chloroquine (24 mg/kg/day × 3 days, see Table 1). Thereafter, additional relapses were treated with the next highest dose of tafenoquine combined with chloroquine at 24 mg/kg/day for three days.

### Radical curative effects of tafenoquine combinations (Cohort 2)

A number of different tafenoquine combination regimens were evaluated in this cohort. Two groups of six animals were given tafenoquine at a dose of 0.6 mg/kg/day × 3 days combined with either chloroquine (24 mg/kg/day × 3) or Coartem^® ^(artemether-lumefantrine 3/18 mg/kg twice per day for three days). A third group of six monkeys were given a single dose of tafenoquine at 12 mg/kg in combination with mefloquine (30 mg/kg administered as two spit doses, six hours apart). There was one chloroquine control group (n = 2) at 24 mg/kg/day × 3 days. A total mefloquine dose of 30 mg/kg was selected on prior unpublished studies demonstrating its efficacy against blood stage *P. cynomolgi*. Mefloquine was administered as two equal 6-hour split doses for logistical reasons and since split dosing is normal clinical practice. A single dose of tafenoquine was used in combination with a split, single day dose of mefloquine, since at the time this study was conducted (2005), this reflected a likely manner in which such a combination would be used in a clinical setting. Coartem is a combination of artemether and lumefantrine (1:6 ratio). Six doses of 12 hours apart are required to effect cure of drug-resistant malaria [17]. Each dose consists of 80/480 mg artemether/lumefantrine (1.1-1.6/6.9-9.6 mg/kg for 50-70 kg person) for a total dose of 480/2880 mg (6.9-9.6/41-58 mg/kg for a 50-70 kg person). The total curative dose of artemether against *P. falciparum *in owl monkeys (*Aotus *sp.) is 24 mg/kg, 3.6 fold higher than the total curative clinical dose. Therefore, in the absence of efficacy data for artemether/lumefantrine in Rhesus monkeys, a scaling factor of approximately two was applied, since this is the usual difference between humans and Rhesus monkeys for most blood schizonticides [9]. Differences in the proportion of animals radically cured on the tafenoquine-mefloquine and tafenoquine-Coartem regimens versus the tafenoquine-chloroquine regimen were tested using Fisher's Exact test [18,19].

### Anti-relapse activity of the minimum curative dose of tafenoquine alone and with chloroquine (Cohort 3)

It was determined whether the efficacy of tafenoquine (total dose of 1.8 mg/kg/day) against hypnozoites was diminished in the absence of chloroquine, by eliminating blood stage parasites prior to treatment. If this was the case, it would imply that the presence of chloroquine was required for tafenoquine to exert an antihypnozoite effect at this dose. Six monkeys were given tafenoquine (0.6 mg/kg/day × 3 days) alone or with chloroquine (24 mg/kg/day × 3 days) after a ten-day course of intramuscular quinine to eliminate blood stage parasites (loading dose of 40 mg/kg base and followed by 20 mg/kg base twice daily for ten days; total dose 420 mg/kg) shown in pilot studies to eliminate blood stage parasites. All monkeys were negative by blood smear at the time of tafenoquine and tafenoquine/chloroquine dosing. The primary endpoint of the study was the proportion of radical cures observed in each group. Differences between groups in terms of cure rates were determined using Fisher's Exact Test.

### Tafenoquine, chloroquine and desethylchloroquine plasma concentrations

Plasma samples were obtained from animals when feasible at 12, 24, 48, 60, 96 and 168 h (after the first dose) in order to measure tafenoquine, chloroquine and desethylchloroquine concentrations (additional samples were not obtained in order to stay below the maximum acceptable blood collection volume under the IACUC protocol). Tafenoquine, chloroquine and desethylchloroquine concentrations were determined simultaneously using HPLC-MS/MS after precipitation of plasma proteins. The lower limits of quantification for all three analytes was determined to be 10 ng/ml, using a 100 μl plasma sample with an upper limit of quantitation of 1000 ng/ml. Plasma concentrations below the lowest standard were considered to be unquantifiable. Quality control (QC) samples prepared at three different analyte concentrations and stored with the study samples, were analyzed with each batch of samples. For the analysis to be acceptable, no more than one-third of the QC results were to deviate from the nominal concentration by more than 15%, and at least 50% of the results from each QC concentration had to be valid.

### Measurement of methaemoglobin and other hematology and blood chemistry endpoints

Changes in methaemoglobin levels were monitored using a Radiometer OSM3 Hemoximeter as previously described [20]. Methaemoglobin levels were determined on Days 0, 1, 2, 4 and 7 post-treatment. Other endpoints including haematocrit, haemoglobin and complete blood counts were determined on Day 0, daily from Days 6-90 and twice weekly thereafter until conclusion of the experiment. Changes in these endpoints were consistent with the effects of malaria or recovery after drug treatment. No drug-attributable changes in these endpoints (other than methaemoglobin levels) were observed.

## Results

### Patency and relapse patterns amongst *P. cynomolgi*-infected rhesus monkeys and utility of different chloroquine regimens (Cohorts 1-3)

In the present series of three cohorts of monkeys, representing fifty four animals, patency was consistently observed in all cases on Day 8.

In Cohort 1, one control monkey receiving 16 mg/kg/day chloroquine experienced a delay in clearance of the primary infection. At the time of the study, and in the absence of chloroquine and desethyl-chloroquine concentrations which were determined later, the research team was concerned that this might represent a failure of the three day regimen. Therefore, the chloroquine dose was increased to 24 mg/kg/day for three days from 16 mg/kg/day for three days in an attempt to suppress delays in primary infection.

The 24 mg/kg/day × 3 regimen cleared parasitaemia in all (n = 10) instances in which it was given to control animals to treat relapses, and resulted in radical cure in all (n = 9) instances in which it was administered in combination with the standard curative seven days of primaquine (at experiment termination, see Tables 1 and Additional File 1). A transient increase in parasitaemia prior to clearance was observed in a single instance. Average times to the first and second relapses from the last day of chloroquine treatment were 9.8 +/- 1.7 and 12.7 +/- 0.8 days (Table 1).

### Radical curative effects of tafenoquine at various doses alone and in combination with chloroquine (Cohort 1)

When given alone, the minimum dose of tafenoquine required to radically cure 2 of 2 monkeys was 6 mg/kg/day × 3 days (Additional File 1 and Table 2). When given with chloroquine at 16 mg/kg/day × 3 days, the minimum curative dose of tafenoquine was 0.6 mg/kg/day × 3 days (Additional File 1 and Table 2). In monkeys given 0.6 mg/kg/day or 0.2 mg/kg/day tafenoquine alone for three days, parasitaemia continued to rise after administration of the drug indicating early blood-stage treatment failure. Overall these data suggest that the addition of chloroquine decreased the dose of tafenoquine required for radical cure by up to ten-fold.

**Table 2:** Summary of Experimental Outcomes for Different TQ treatments in Cohort 1.

TQ Daily Dose	TQ Alone	TQ + CQ (16 mg/kg/day × 3)
**(mg/kg/day × 3)**	**#cured/not cured (%)**	**#cured/not cured (%)**

0.06	-	0/2 (0)
0.2	0/2 (0)*	0/2 (0)
0.6	0/2 (0)*	2/0 (100)
2	1/1 (50)	2/0 (100)
6	2/0 (100)	-

* Early treatment failures

**Figure 1:**
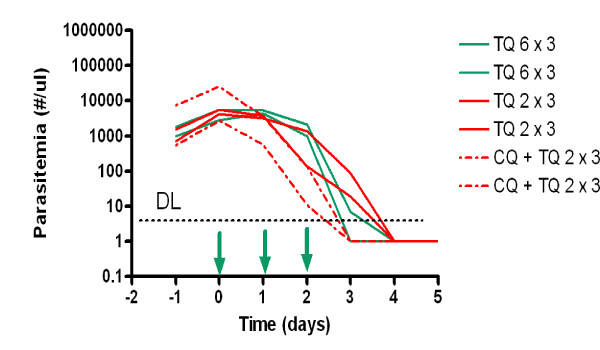
**Course of parasitaemia in Cohort 2 monkeys given tafenoquine (TQ) at 6 mg/kg base/day or 2 mg/kg base/day alone or in combination with 16 mg/kg chloroquine base/day for three days**. The abbreviation DL refers to the detection limit of parasitaemia by microscopy. For the purposes of illustration parasitaemia levels falling below the limit of detection (approximate location indicated by hashed line) were arbitrarily assigned a value of 1 parasite/μl. Arrows indicate drug administration.

There was an apparent pharmacodynamic interaction between chloroquine and tafenoquine against blood stage parasites. Tafenoquine administered alone exhibited an apparent dose-related effect on parasite clearance time (Figure 1). In monkeys given tafenoquine monotherapy at doses of 2 and 6 mg/kg/day for three days, average parasite clearance times were 4 and 3.5 days respectively (Figure 1). This compares to a parasite clearance time of 3.5 days after a 1200 mg dose of tafenoquine against *P. vivax *in humans [2]. Lower doses of tafenoquine alone did not clear parasitaemia in this study. Chloroquine decreased parasite clearance time to 3 days when coadministered with tafenoquine at 2 mg/kg/day for three days. At lower doses of tafenoquine, chloroquine administered after initial tafenoquine failure eventually resulted in radical cure. These data suggest that chloroquine compensates for the poorer blood schizonticidal effects of lower tafenoquine doses.

**Figure 2:**
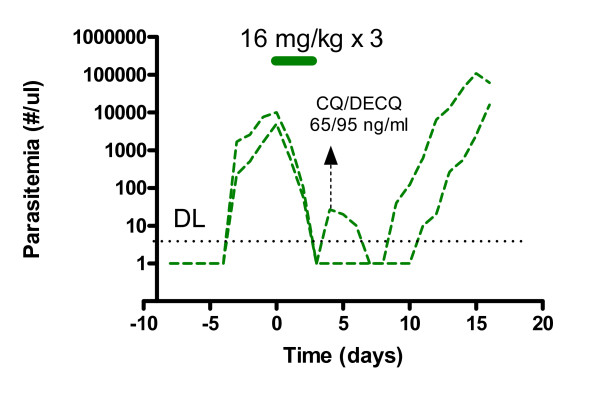
**The course of infection in the two Cohort 2 control monkeys given chloroquine alone at 16 mg/kg/base/day for three days**. For the purposes of illustration parasitaemia levels falling below the limit of detection were arbitrarily assigned a value of 1 parasite/μl. The transient increase in parasitaemia in one of the monkeys after initial clearance is interpreted to be a delay in the primary attack. The concentrations of chloroquine and desethylchloroquine observed at the time of peak parasitaemia in one monkey are indicated.

Chloroquine-cleared parasitaemia in the control monkeys (Figure 2). However, in one monkey a transient increase in parasitaemia was observed after an initial clearance prior to eventual clearance (Figure 2). Similar transient increases were observed prior to eventual clearance in some of the monkeys treated with chloroquine and 0.2 or 0.06 mg/kg/day of tafenoquine for three days. These instances were interpreted to be delays in the primary attack, emerging from late tissue primary schizont development from delayed sporozoite infection. In the control animal where such a transient increase in parasitaemia occurred, the maximum observed chloroquine and desethylchloroquine concentrations were 165 and 1140 ng/ml (516 and 3800 nM) respectively and at the time of the transient increase in parasitaemia were 65 and 95 ng/ml (204 and 315 nM) respectively. In clinical use, peak chloroquine and desethylchloroquine concentrations were 200-2000 and 90-1000 nM respectively [21], suggesting that in this particular animal chloroquine and desethyl chloroquine concentrations approximated those observed clinically.

In this cohort tafenoquine was also administered at a single dose of 12 mg/kg PO. This regimen cleared parasitaemia in both monkeys (Additional File 1), and radically cured one of the two monkeys.

### Radical curative effects of tafenoquine combinations (Cohort 2)

The efficacy of two alternate regimens, mefloquine and artemether-lumefantrine, in combination with tafenoquine were investigated. Five of five monkeys given tafenoquine-chloroquine (the sixth monkey was excluded for reasons unrelated to study drug) and five of six monkeys given tafenoquine-lumefantrine-artemether were radically cured (Additional File 1). This difference in cure rates was not significant (Fisher's exact test, *P *= 0.54). A cure rate of 100% (6 of 6 monkeys) was observed in the mefloquine-tafenoquine group (Additional File 1). Chloroquine alone (24 mg/kg/day for three days) cleared (without transient increases) parasitaemia in both the control monkeys with subsequent relapses.

### Anti-relapse activity of the minimum curative dose of tafenoquine alone and with chloroquine following elimination of blood stages with quinine (Cohort 3)

Radical cures were achieved in 6 of 6 monkeys given tafenoquine alone (0.6 mg/kg/day for three days) and 6 of 6 monkeys given tafenoquine at the same dose combined with chloroquine (24 mg/kg/day for three days) following quinine treatment. Thus, the efficacy of anti-relapse activity of 1.8 mg/kg tafenoquine is not dependent on co-administration with chloroquine.

#### Overall efficacy of tafenoquine at its minimum curative dose

The overall efficacy of the presumed minimum curative dose of tafenoquine is summarized in Table 3. When the regimens are considered collectively, tafenoquine was 100, 95 and 93% effective when administered alone (after quinine pretreatment), combined with chloroquine at low or high doses, or any blood schizontocidal, respectively. Thirty three of thirty five treatments at this dose level were successful, for an overall cure rate of 94%.

**Table 3:** Anti-relapse efficacy of a total tafenoquine dose 1.8 mg/kg (0.6 mg/kg/day for three days or 1.8 mg/kg once) monotherapy or in combination with chloroquine and other blood stage regimens.

Treatment Cohort	Notes of clarification	Outcome #cured/total (%)
TQ Alone*		
Cohort 3	TQ given alone after quinine pretreatment	6/6 (100)
**TQ + CQ 16**		
Cohort 1**	CQ given sequentially after blood stage failure of first tafenoquine treatment regimen	2/2 (100)
Cohort 2	CQ + TQ given simultaneously for first treatment	2/2 (100)
**TQ + CQ 24**		
Cohort 1	CQ given sequentially after blood stage failure of first relapse treatment	2/2 (100)
Cohort 1	Treatment of second relapse	1/2 (50)
Cohort 2	CQ + TQ given simultaneously for first treatment	5/5 (100)
Cohort 3	TQ given with CQ after quinine pretreatment to eliminate blood stage parasites	6/6 (100)
Unpublished data	TQ given as a single dose for first treatment (data not shown)	2/2 (100)
**TQ + Other Anti-malarials**		
Cohort 2	Simultaneous combination with artemether-lumefantrine for initial treatment	5/6 (83)
Unpublished data	Simultaneous combination with atovaquone-proguanil for initial treatment (data not shown)	2/2 (100)
**Total **		
Tafenoquine alone		6/6 (100)
TQ + CQ		20/21 (95)
TQ + all BS**All		27/29 (93)
TQ		33/35 (94)

* This refers to published data in which blood stage parasites were eliminated prior to tafenoquine or tafenoquine/chloroquine treatment. ** These are monkeys (Table 1) which were treated with chloroquine after tafenoquine monotherapy failed to clear blood stage parasites. For the purposes of the anti-relapse endpoint, these monkeys were considered to have been given a combination regimen. ** All blood schizonticidal drugs.

### Methaemoglobin and tafenoquine plasma concentrations

**Figure 3:**
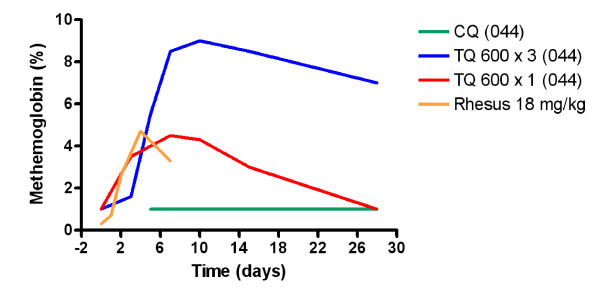
**Methaemoglobin levels in Rhesus monkeys given a total dose of 18 mg/kg (6 mg/kg/day × 3) tafenoquine and human subjects given chloroquine (CQ), a single 600 mg or three 600 mg doses of tafenoquine (TQ)**. Clinical data are from Walsh et al (2004).

**Figure 4:**
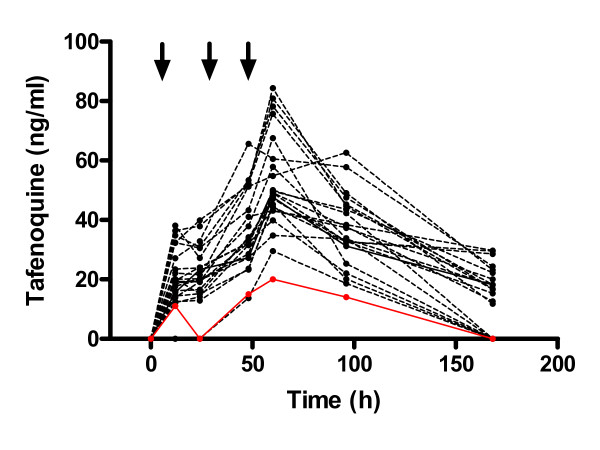
**Tafenoquine plasma concentration time curves for 22 individual monkeys given tafenoquine at a dose of 0.6 mg/kg/day for three days under various conditions**. No other data for tafenoquine plasma levels for tafenoquine were available at any other dose. The arrows indicate the times of dosing. Maximum observed tafenoquine plasma concentrations were between 20-85 ng/ml between 48 and 96 h after dosing. Data were unavailable for the two monkeys that relapsed at this dose. Data (red circles and solid line) were available for only one of two monkeys in Cohort 2 where this dose given alone for radical cure failed in both animals, and chloroquine rescue was required.

Tafenoquine induced a dose-related increased in methaemoglobin from baseline (data not shown). At the highest dose of tafenoquine, methaemoglobin levels peaked at 4.7% (Figure 3). In contrast in humans peak methaemoglobin was 4.5% after a 600 mg dose and 9.0% after an 1800 mg dose. Tafenoquine plasma-concentration time curves after administration of 0.6 mg/kg/day (the minimum anti-relapse dose) for three days in n = 22 (for which data were available) under various conditions are presented in Figure 4. Data were not able to be generated for other doses of tafenoquine. The maximum observed tafenoquine concentrations clustered narrowly between 20 and 84 ng/ml at 48-96 h post-dosing. In two clinical *P. vivax *cases where tafenoquine was given in a total dose of 1200 mg over three days and resulted in radical cure, maximum observed plasma concentrations were 550 and 1100 ng/ml at 48 h [2]. Plasma tafenoquine levels were unavailable from the two instances where relapses occurred at this dose. Plasma tafenoquine levels were only available from one of two animals (Cohort 2) in which this regimen failed to clear blood stage parasites and required chloroquine rescue. Tafenoquine levels were lower in this animal than the others (Figure 4).

## Discussion

In the present study, a modified Rhesus monkey-*P. cynomolgi *model was utilized to evaluate the radical curative efficacy of tafenoquine combined with various blood schizonticidal drugs administered at human equivalent doses. The data show that tafenoquine, when combined with chloroquine and other drugs is an effective radical curative agent against *P. cynomolgi *malaria at much lower doses than those used in prior *P. vivax *clinical trials. In the discussion that follows, some of the methodologic aspects of this animal model are addressed together with potential relevance the data in terms of future clinical use of tafenoquine.

In the present model application, some refinements to the original methods of Schmidt *et al *[9] were made. These involved the methods of preparation of sporozoites prior to inoculation, the use of standard inoculation dose and the use of three-day chloroquine regimens. The more refined methods used for sporozoite processing are likely to have increased the viability of the inoculums given. This may explain the greater variability in the time to patency observed by Schmidt *et al *[9] compared to this study (average time to patency in days +/- standard deviation of 8.4 +/- 0.6 days, n = 538 v 8.0 +/- 0.0 days, n = 54). The utility of the three-day chloroquine regimens is evidenced by 100% clearance in all instances (n = 11) when either regimen was used to treat relapses in control monkeys, and radical cure in all (total n = 30) instances but one in which either of the three-day chloroquine regimens were given in combination with effective regimens of tafenoquine or primaquine for radical cure. These observations suggest that the three-day chloroquine regimens can be used in place of seven-day regimens.

Occasional increases in parasitaemia prior to clearance after treatment with the three-day chloroquine regimens were observed. When this first occurred in the Cohort 1 controls, the research team was concerned that these may have been failures, so the chloroquine dose was increased. However other data suggest this is a feature of the animal model itself. Usually, when chloroquine is given as a seven-day regimen, a dose of 10 mg/kg/day is the usual choice. This is based on the observation that lower doses are sub-curative [13]. However, in some instances where this regimen is administered for the primary treatment, parasitaemia is not initially cleared. In such cases the course of treatment is extended for 2-4 days, but sometimes up to 7 additional days until blood stage parasites are cleared [14]. This phenomenon occurs periodically in some experiments and is presumably related to the large sporozoite inoculums given. Since effective levels of chloroquine and desethylchloroquine were present in the two control monkeys given 16 mg/kg/day for three days it is likely that the transient increase in parasitaemia observed in one of these monkeys represents the normal pattern of variability in the model, rather than a failure of the three-day chloroquine regimen.

The minimum dose of tafenoquine required for prevention of *P. vivax *relapses in humans has not been definitely established, despite the execution of two clinical studies to determine this. Walsh *et al *[22,23] evaluated the protective efficacy of tafenoquine relative to chloroquine alone, and when administered sequentially following treatment of symptomatic *P. vivax *malaria with chloroquine (Table 4). Several factors suggest caution should be exercised when interpreting these human data. The group sizes were small, and protective efficacy calculations were made based on single relapses. While care was taken to prevent re-infection, these cannot be ruled out in the endemic area situation. No dose-effect relationship is clear, and the confidence intervals across the treatment groups were wide and overlapped. The nature of the relapses (delayed as is the case for regional 'primaquine-tolerant *P. vivax *malaria' or associated with vomiting) means that they cannot be unequivocally attributed to the failure of tafenoquine. All that can be stated definitively is that total doses of tafenoquine greater than or equal to 500 mg appear to prevent relapses in most instances. In the same studies, primaquine (15 mg/day) was administered for 14 days in a controlled setting yielding a protective efficacy of 80%. Tafenoquine at a dose level that produced this level of anti-relapse efficacy would still be useful, and would likely have greater effectiveness in the field due to the much more convenient dosing regimen. The available clinical data are not helpful in determining what this dose might be.

**Table 4:** Anti-relapse efficacy of tafenoquine in various regimens and primaquine in human cases compared to chloroquine in Thailand (from Walsh et al 1999; 2004).

Drug	Dose	Total Dose	#Subjects	#Relapses	Efficacy (% +/- 95% CI)	Comment on Relapse
						
Tafenoquine	500 mg	500	9	1	87 (-37-100)	Relapse delayed
Tafenoquine	600 mg	600	18	1	96 (67-99)	Vomiting
Tafenoquine	600 mg × 3	1800	19	0	100 (78-100)	
Tafenoquine	300 mg × 7	2100	19	0	100 (78-100)	
Tafenoquine	300 mg × 7	2100	15	0	100 (24-100)	
Tafenoquine	500 mg × 3, repeated	3000	11	1	87 (-34-100)	Relapse delayed
Primaquine	15 mg × 14		12	3	80 (15-97)	

In the present studies, it was shown that when tafenoquine is combined with chloroquine, the doses of tafenoquine (total minimum curative dose of 1.8 mg/kg) required for radical cure can be reduced by up to ten-fold compared to administration of the drug alone (total MCD 18 mg/kg) in a relapsing malaria model. At such a low dose of tafenoquine, plasma levels of tafenoquine were substantially lower than those observed after a 1,200 mg dose in humans (Figure 4,[2]). Based on approximately equivalent methaemoglobin responses, the Rhesus monkey MCD of 18 mg/kg tafenoquine is likely equivalent to a dose of 600 mg in humans [23]. Based on approximately equivalent parasite clearance times, the Rhesus monkey MCD of tafenoquine of 18 mg/kg when administered alone is likely equivalent to a dose of 1,200 mg in humans [2]. These data provide the basis for proposing the hypothesis that much lower tafenoquine doses may impart substantial anti-relapse activity in future clinical studies. Administration of such low doses may be required if the liability of haemolytic toxicity in G6PD deficiency humans is to be mitigated.

This dose reduction does not appear to specifically require chloroquine. Although one relapse/recrudescence was observed in the artemether-lumefantrine group, no statistical difference in efficacy was shown between tafenoquine-chloroquine and tafenoquine-artemether-lumefantrine combination regimens (both with tafenoquine at a total dose of 1.8 mg/kg). The single failure in the tafenoquine-artemether-lumefantrine group was attributable to chance, since overall the observed probability of a relapse with 1.8 mg/kg tafenoquine is approximately 6% (Table 4). At the same dose of tafenoquine, it was also shown that there was no difference in anti-relapse efficacy when the compound was administered alone or with chloroquine after quinine pretreatment to eliminate blood stage parasites. This observation and the approximate equivalence of the tafenoquine-chloroquine and tafenoquine-artemether-lumefantrine regimens suggest that tafenoquine can be combined with any effective blood schizonticidal drug. Tafenoquine at a single dose of 12 mg/kg PO cured only one of two animals when administered as monotherapy, a failure we interpret to be a recrudescence rather than a relapse, since much lower doses were effective for anti-relapse therapy. However, the same dose of tafenoquine was very effective when combined with mefloquine (six of six animals cured). The effectiveness of the combination demonstrates the potential utility of single day tafenoquine combination regimen, although the use of therapeutic level doses of mefloquine would require very careful thought, and would only be justifiable in the appropriate risk-benefit context (i.e. elimination attempts).

The ability to lower tafenoquine doses with a partner blood schizonticide comes at a cost. It was clear in Cohort 1 monkeys that chloroquine improved parasite clearance times, and was required for adequate elimination of blood stage parasites at lower tafenoquine doses lacking useful blood schizonticidal activity. Thus, if chloroquine was proposed as the partner drug, and the tafenoquine dose was lowered to mitigate the risk of haemolytic toxicity (and, therefore, its blood schizontocidal effect was reduced), it is possible that any putative synergy of tafenoquine and chloroquine against the blood stages of chloroquine-resistant *P. vivax *would be lost. The alternative would be to employ an alternate blood-stage regimen that is fully effective in its own right against blood stage *P. vivax *(and preferably also *P. falciparum*). Artemether-lumefantrine would serve this purpose as would other drugs in clinical use and development (e.g. piperaquine-dihydroartemisinin). Alving *et al *[24] showed that cure rates of sub-curative doses of primaquine could be increased if combined with chloroquine or quinine. This is also a possibility with tafenoquine, and was a question left incompletely addressed by this study. If this does occur it is insufficient to result in a substantial reduction in the dose required for anti-relapse efficacy since a dose of 0.2 mg/kg/day of tafenoquine for three days combined with chloroquine is not as efficacious as 0.6 mg/kg/day tafenoquine alone for three days. These observations should be considered carefully by clinicians planning future tafenoquine-*P. viva*x clinical trials.

## Competing interests

Geoffrey Dow, Simon Cozens, David Kenworthy Anne Miller, Jim Veazey and Colin Ohrt are all past or present members of and/or support the US Army and/or GSK clinical teams developing tafenoquine for the P. vivax radical cure and/or prophylaxis indications.

## Authors' contributions

GD, DKY, WM, CO, JV and MG contributed to the design of experiments. MG led their execution. HP, JK, RI and SK made substantial contributions to the execution of the animal studies. SC, DKE, and AM provided input on appropriate sample collections times for the PK studies and provided plasma drug levels. GD wrote the manuscript. The contribution of all authors was essential for successful execution of the study. All authors read and approved the final version of the manuscript.

## Supplementary Material

Additional file 1 **Summary of treatment outcomes in three cohorts of Rhesus monkeys dosed with tafenoquine and other antimalarials**. The file provides a summary of treatment outcomes in three cohorts of Rhesus monkeys dosed with tafenoquine and other antimalarials. Click here for file
