# Supplementary material for: Radical curative efficacy of tafenoquine combination regimens in Plasmodium cynomolgi-infected Rhesus monkeys (Macaca mulatta)
Source: Malar J. 2011 Jul 29;10:212. doi: 10.1186/1475-2875-10-212 (PMC3161915; doi:10.1186/1475-2875-10-212)
Supplement: Additional file 1 — Summary of treatment outcomes in three cohorts of Rhesus monkeys dosed with tafenoquine and other antimalarials. The file provides a summary of treatment outcomes in three cohorts of Rhesus monkeys dosed with tafenoquine and other antimalarials. [file 1475-2875-10-212-S1.DOC]

**Summary of Treatment Outcomes in Cohort 1 to 3 Monkeys.**

| **Monkey #** | **Treatment^1^** | **Relapse Day After Treatment^1^** | **First Relapse Treatment^1^** | **Relapse Day After Treatment** | **Second Relapse Treatment^1^** | **Relapse Day After Treatment** | **Third Relapse Treatment^1^** |
| --- | --- | --- | --- | --- | --- | --- | --- |
| **COHORT 1**  R305  R316  R304  R313  R227  R326  R216  R306  R257  R259  R251  R308  R232  R318  R202  R211  R301  R309  R220  R302  **COHORT 2**  R124  R235  R113  R143  R210  R225  R233  R252  R122  R209  R212  R214  R237  R238  R116  R223  R234  R236  R240  R241  **COHORT3**  R332  R340  R256  R310  R323  R324  R337  R338  R228  R245  R317  R322  R325  R333 | CQ 16x3  CQ 16 x3  TQ 6.0x3  TQ 6.0x3  TQ 2.0x3  TQ 2.0x3  TQ 0.6x3 then CQ 16x3^2^  TQ 0.6x3 then CQ 16x3^2^  TQ 0.2x3 then CQ 16x3^2^  TQ 0.2x3 then CQ 16x3^2^  CQ 16x3+TQ 2.0x3  CQ 16x3+TQ 2.0x3  CQ 16x3+TQ 0.6x3  CQ 16x3+TQ 0.6x3  CQ 16x3+TQ 0.2x3  CQ 16x3+TQ 0.2x3  CQ 16x3+TQ 0.06x3  CQ 16x3+TQ 0.06x3  TQ 12x1  TQ 12x1  CQ 24x3  CQ 24x3  TQ 0.6+CQ 24x3  TQ 0.6+CQ 24x3  TQ 0.6+CQ 24x3  TQ 0.6+CQ 24x3  TQ 0.6+CQ 24x3  TQ 0.6+CQ 24x3  TQ 12+MQ 30x1  TQ 12+MQ 30x1  TQ 12+MQ 30x1  TQ 12+MQ 30x1  TQ 12+MQ 30x1  TQ 12+MQ 30x1  TQ 0.6x3+COARTEM  TQ 0.6x3+COARTEM  TQ 0.6x3+COARTEM  TQ 0.6x3+COARTEM  TQ 0.6x3+COARTEM  TQ 0.6x3+COARTEM  CQ 24x3  CQ 24x3  QUIN then TQ 0.6x3  QUIN then TQ 0.6x3  QUIN then TQ 0.6x3  QUIN then TQ 0.6x3  QUIN then TQ 0.6x3  QUIN then TQ 0.6x3  QUIN then TQ 0.6x3+CQ 24x3  QUIN then TQ 0.6x3+CQ 24x3  QUIN then TQ 0.6x3+CQ 24x3  QUIN then TQ 0.6x3+CQ 24x3  QUIN then TQ 0.6x3+CQ 24x3  QUIN then TQ 0.6x3+CQ 24x3 | 10  7  None  None  None  19  None  None  5  34  None  None  None  None  2^3^  13^3^  7^3^  8^3^  None  9  9  12  None  None  None  Withdrawn  None  None  None  None  None  None  None  None  None  None  18  None  None  None  8  10  None  None  None  None  None  None  None  None  None  None  None  None | CQ 24x3  CQ 24x3  -  -  -  TQ 6x3  -  -  TQ 0.6x3 then CQ 24x3^2^  TQ 0.6x3 then CQ 24x3^2^  -  -  -  -  CQ 24x3+TQ 0.2x3  CQ 24x3+TQ 0.2x3  CQ 24x3+TQ 0.06x3  CQ 24x3+TQ 0.06x3  -  CQ 10 x 7+PQ  CQ 24 x 3  CQ 23 x 3  -  -  -  -  -  -  -  -  -  -  -  -  -  -  COARTEM x 3+PQ  -  -  -  CQ 24 x 4  CQ 24 x 3  -  -  -  -  -  -  -  -  -  -  -  - | 12  13  -  -  -  None  -  -  None  None  -  -  -  -  11  17  7  7  -  None  9  10  -  -  -  -  -  -  -  -  -  -  -  -  -  -  None  -  -  -  9  12  -  -  -  -  -  -  -  -  -  -  -  - | CQ 24x3+PQ  CQ 24x3+PQ  -  -  -  -  -  -  -  -  -  -  -  -  CQ 24x3+TQ 0.6x3  CQ 24x3+TQ 0.6x3  CQ 24x3+TQ 0.2x3  CQ 24x3+TQ 0.2x3  -  -  CQ 24 x 3+PQ  CQ 24 x 3+PQ  -  -  -  -  -  -  -  -  -  -  -  -  -  -  -  -  -  -  CQ 24 x 3+PQ  CQ 24 x 3+PQ  -  -  -  -  -  -  -  -  -  -  -  - | None  None  -  -  -  -  -  -  -  -  -  -  -  -  19  None  16  16  -  -  None  None  -  -  -  -  -  -  -  -  -  -  -  -  -  -  -  -  -  -  None  None  -  -  -  -  -  -  -  -  -  -  -  - | -  -  -  -  -  -  -  -  -  -  -  -  -  -  CQ 24 x 3+ PQ^4^  -  CQ 24 x 3+ PQ^4^  CQ 24 x 3+ PQ^4^  -  -  -  -  -  -  -  -  -  -  -  -  -  -  -  -  -  -  -  -  -  -  -  -  -  -  -  -  -  -  -  -  -  -  -  - |

^1^ All treatments listed in the format drug XXxY, where the drug us abbreviated as indicated XX refers to the daily dose in mg/kg/day and Y refers to the number of days of dosing. Drugs were administered orally, once daily unless otherwise indicated. CQ refers to chloroquine, TQ refers to tafenoquine, QUIN refers to quinine, COARTEM refers to artemether-lumefantrine, MQ refers to mefloquine, and PQ refers to primaquine. The dose of primaquine was always 1.78 mg/kg/day for seven days. The dose of artemether/lumefantrine was 3/18 mg/kg twice per day for three days. The dose of mefloquine was 30 mg/kg administered as two evenly divided doses 6 h apart. Quinine was given intramuscularly as a total dose of 420 mg/kg administered over 10 days. Where a relapse treatment is ‘NONE’ this means that the prior primary or relapse treatment resulted in radical cure and no relapse has occurred. ^2^ Chloroquine was administered because the indicated tafenoquine regimen resulted in early treatment failure. ^3^ In these monkeys, initial clearance of parasitemia was followed by a transient increase above the detection limit followed by clearance and subsequent relapse. The day of eventual relapse is indicated. For monkey R202, parasitemia cleared on the first day after treatment then recrudesced on Day 2. ^4^ Animals were cured. ^5^ Quinine was administered first to eliminate asexual blood stages followed by tafenoquine alone or tafenoquine and chloroquine.
